# Effects of climate change on different geographical populations of the cotton bollworm *Helicoverpa armigera* (Lepidoptera, Noctuidae)

**DOI:** 10.1002/ece3.8426

**Published:** 2021-12-06

**Authors:** Jian Huang

**Affiliations:** ^1^ Institute of Desert Meteorology China Meteorological Administration Urumqi China; ^2^ Central Asian Research Center for Atmospheric Sciences Urumqi China

**Keywords:** abrupt change, climate change, geographical population, *Helicoverpa armigera*

## Abstract

The effects of climate change on pest phenology and population size are highly variable. Understanding the impacts of localized climate change on pest distribution and phenology is helpful for improving integrated pest management strategies. Here, the population dynamics of cotton bollworms (*Helicoverpa armigera*) from Maigaiti County, south Xinjiang, and Shawan County, north Xinjiang, China, were analyzed using a 29‐year dataset at lower latitudes and a 23‐year dataset at higher latitudes to determine the effects of climate change on the population dynamics of *H*. *armigera*. The results showed that all generations of *H*. *armigera* at both sites showed increasing trends in population size with climate warming. Abrupt changes in phenology and population number occurred after abrupt temperature changes. Climate change had a greater effect on the phenology of *H*. *armigera* at higher latitudes than at lower latitudes and led to a greater increase in population size at lower latitudes than at higher latitudes; the temperature increase at higher latitudes will cause a greater increase in the adult moth population size in the future compared to that at lower latitudes; and abrupt changes in the phenology, temperature increase, and population size at lower latitudes occurred earlier than those at higher latitudes. Thus, it is necessary to develop sustainable management strategies for *Helicoverpa armigera* at an early stage.

## Graphical abstract

Climate change had a greater effect on phenology of *Helicoverpa armigera* at higher latitude than that at lower latitude; climate change caused a more increase of population size at lower latitude than that at higher latitude; the accumulated temperature (AT) percentage at higher latitude had a rapid increase and cause adult moth increase in the future than those at lower latitude; the abrupt changes of phenology, AT and population size at lower latitude were earlier than those at higher latitude. Thus, it is necessary to early make relative strategies to manage the insect pest.

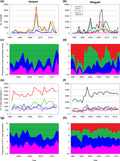

## 1 INTRODUCTION

Climate change is an indubitable fact that has had a profound influence on the biological behaviors of terrestrial organisms, such as changing organism distributions (Franco et al., 2006; Parmesan, 1996; Parmesan et al., 1999; Parmesan & Yohe, 2003), phenology (Huang & Li, 2015; Parmesan, 2007; Satake et al., 2006; Westgarth‐Smith et al., 2007), overwintering survival rates (Bale et al., 2002; Huang, 2016), and occurrence dynamics (Jepsen et al., 2011; Pelini et al., 2010). As ectotherms, insects, especially insects with a lower thermal tolerance, are highly sensitive to temperature changes (Deutsch et al., 2008).

The cotton bollworm *Helicoverpa armigera* (Hübner) (Lepidoptera: Noctuidae) is one of the most detrimental insect pests in the world (Fitt, 1989). In China, *H*. *armigera* can produce three (Wu, 1999; Zhang et al., 2013), four (Zhai et al., 1992; Zhang et al., 2013), five (Zhang et al., 2000), and six generations (Meng et al., 1962) in a year due to the different geographical environments. The Xinjiang Uygur Autonomous Region (XUAR) is in northwestern China and famous for its high‐quality cotton. XUAR produced 87.3% of China's cotton in 2020 (National Bureau of Statistics, 2020). Therefore, it is necessary to understand the drivers of the population dynamics of *H*. *armigera* in this region.

The phenology or seasonal development of an insect is coordinated with the local environmental conditions. *H*. *armigera* at higher latitudes has a lower development threshold temperature, higher diapause ratio, and longer development duration than those at lower latitudes (Chen & Tu, 2016; Shimizu & Fujisaki, 2006; Wu & Guo, 1997). However, whether this observed response to climatic change represents an evolutionary (genetic) response to selection for efficient breeding or an expression of phenotypic plasticity has not been clarified. When climate warming occurs, the phenology of *H*. *armigera* changes (Huang & Hao, 2018; Huang & Li, 2015). Combined with the changes in host phenology and host varieties, the number of *H*. *armigera* fluctuates sharply (Feng et al., 2010; Nyabo, 1988). Thus, climate change causes significant changes in the phenology and population size of *H*. *armigera*. However, whether climate has the same influence on the phenology and population sizes of *H*. *armigera* in different geographical environments, especially at different latitudes, has not been clarified.

*Helicoverpa armigera* requires sufficient heat accumulation for development. Different temperatures lead to different developmental rates (Wu et al., 1980), inappropriate temperatures cause diapause (Wu & Guo, 2005), and greater accumulated temperature (AT) can increase the emergence rate (Mirondis et al., 2010). Thus, appropriate climate conditions can cause outbreaks of *H*. *armigera* (Dai & Guo, 1994). Populations of *H*. *armigera* at different latitudes have different heat and photoperiods, which leads to populations of *H*. *armigera* having different development rates (Chen & Tu, 2016). For *H*. *armigera*, which can produce several generations in a year, whether the heat of each generation is the same had not been determined and how the change in heat affects the number of adult moths in the context of climate change is poorly understood.

The climate system is nonlinear and discontinuous, and climate change can be abrupt and may dramatically change from one stable status to another, meaning that climate status varies spatiotemporally from one statistical characteristic to another (Fu & Wang, 1992). Thus, it is necessary to analyze and understand abrupt climate change using nonlinear theories and methods, such as the theory of abrupt changes and corresponding detection methods (Yan et al., 2003). The Mann–Kendall test has the merits of a broad detection range, a small artificial impact, and a high degree of quantitativeness (Wei, 1999). Therefore, the Mann‐Kendall test was used to detect abrupt changes in various variables in this study. Detailed theories are provided by Fu and Wang (1992) and Wei (2007). An analysis of the effects of abrupt climate change on climate factors and insects is helpful for understanding changes in population dynamics. However, the effects of abrupt climate change on adult moths of *H*. *armigera* at different latitudes are unknown.

Climate change is highly variable and localized, and the effect of climate change on populations of *H*. *armigera* at different latitudes may present highly variable and localized characteristics. Thus, the objects of this study were to (a) analyze differences in seasonal phenology at different latitudes; (b) compare the number of adult moth changes and analyze the drivers at the two sites; and (c) analyze the effects of accumulated heat at the two sites on the population size and analyze the effects of climate abrupt change on population sizes.

## 2 MATERIALS AND METHODS

### 2.1 Study sites

**FIGURE 1:**
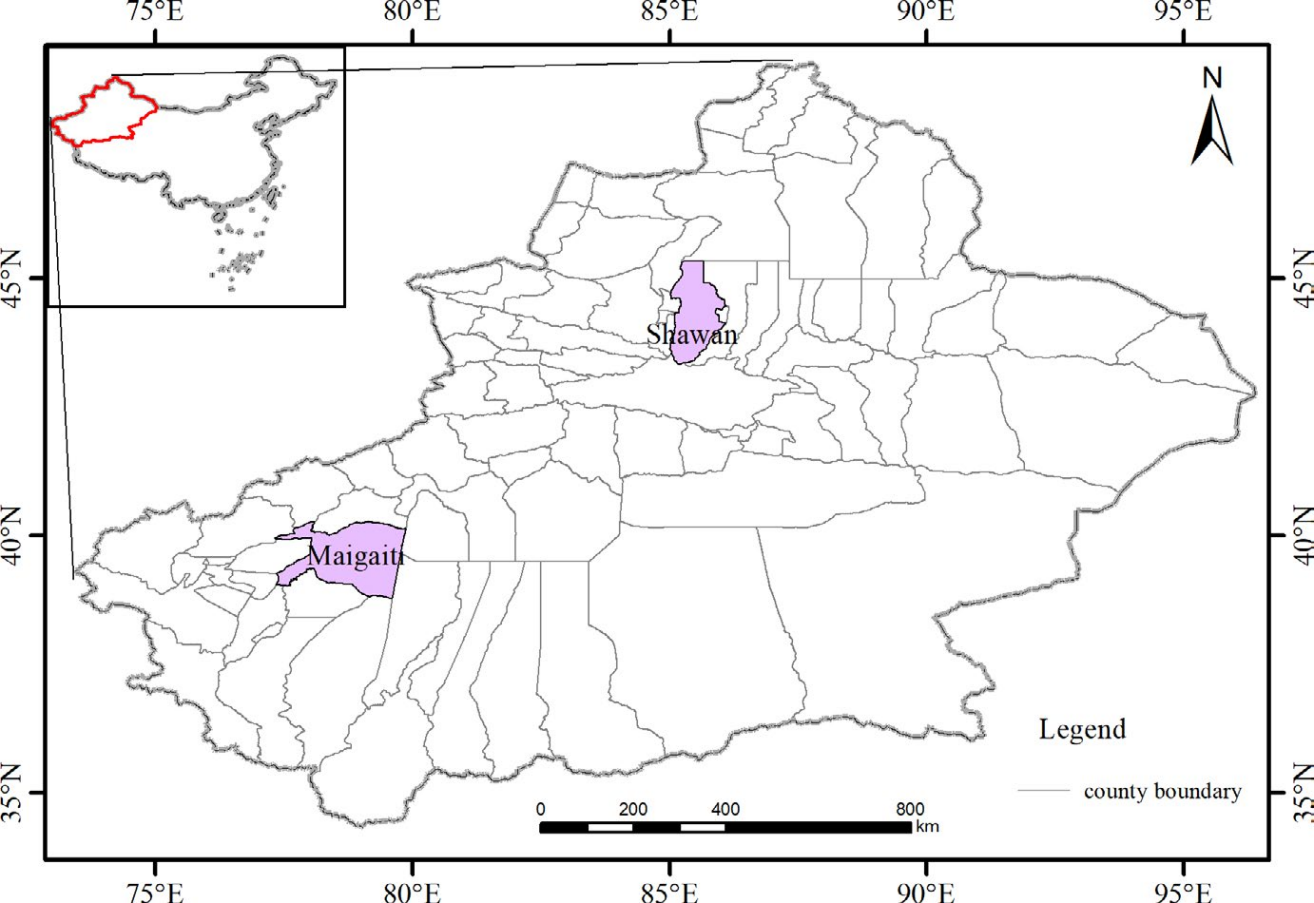
Study sites of Maigaiti and Shawan

Maigaiti County (77° 28′–79°05′E, 38°25′–39°22′N, and 1,155–1,195 m above sea level) (Figure 1) has a total area of 11,023 km^2^ and lies in the western Tarim Basin, XUAR, China (Zhang & Zhang, 2006), and it includes a town and several villages but has no hills or mountains. The area has a temperate continental arid climate, and the annual mean temperature (*T*_mean_), annual mean precipitation, sunshine hours, and mean frost‐free period are 11.7°C, 48.2 mm, 2,806 h, and 214 days, respectively (Zhang & Zhang, 2006). *H*. *armigera* at this site can produce four generations for a year: the overwintering generation (G0), the first generation (G1), the second generation (G2), and the third generation (G3).

Shawan County (84°56′–86°08′E, 43°19′–45°55′N, and 1,290–3,867 m above sea level) (Figure 1) has a total area of 13,100 km^2^ and lies in the southern Junggar Basin, XUAR, China (Zhang & Zhang, 2006), and it includes a town and several villages. The Tianshan Mountains lie in southern Shawan County. The area has a temperate continental arid climate and vertical climatic zone, and the annual mean temperature (*T*_mean_), annual mean precipitation, sunshine hours, and annual mean frost‐free period are 6.6°C, 190 mm, 2,835 h, and 180 days, respectively (Zhang & Zhang, 2006). *H*. *armigera* at this site can produce three generations in a year: the overwintering generation (G0), the first generation (G1), and the second generation (G2).

The lower latitude and higher latitude sites present different geographic and climatic characteristics and different *H*. *armigera* living statuses. Adult moths of *H*. *armigera* from both sites were trapped and used to analyze their population changes.

### 2.2 Weather and *Helicoverpa armigera* moth survey data

Weather parameters were recorded at the Maigaiti and Shawan weather administrations, which lie on the edge of the counties. The adult moths were trapped with a 20 W black light lamp (made by Jiaduo Technology, Industry and Trade Company Limited, China), which was placed in an open field at 1.5 m above the ground, with no trees or higher buildings surrounding the lamp. The distance between the weather station and the lamp was approximately 300 m. The lamp was turned off after dawn and turned on after dusk from early April to late September from 1996 to 2018 in Shawan and from early April to late October from 1989 to 2017 in Maigaiti. The lamp was replaced with a new strip lamp each year. All the set criteria followed the national standards (the State Administration of Quality Supervision Inspection and Quarantine of the People's Republic of China and the Standardization Administration of the People's Republic of China, 2009). Cotton fields, winter wheat fields, corn fields, other crop fields, and vegetable fields surrounded the lamps. The adult moths captured using the lamp were counted daily, and every generation of *H*. *armigera* moths was distinguished using standard methods (Lu et al., 2013; Zhang et al., 2013).

**FIGURE 2:**
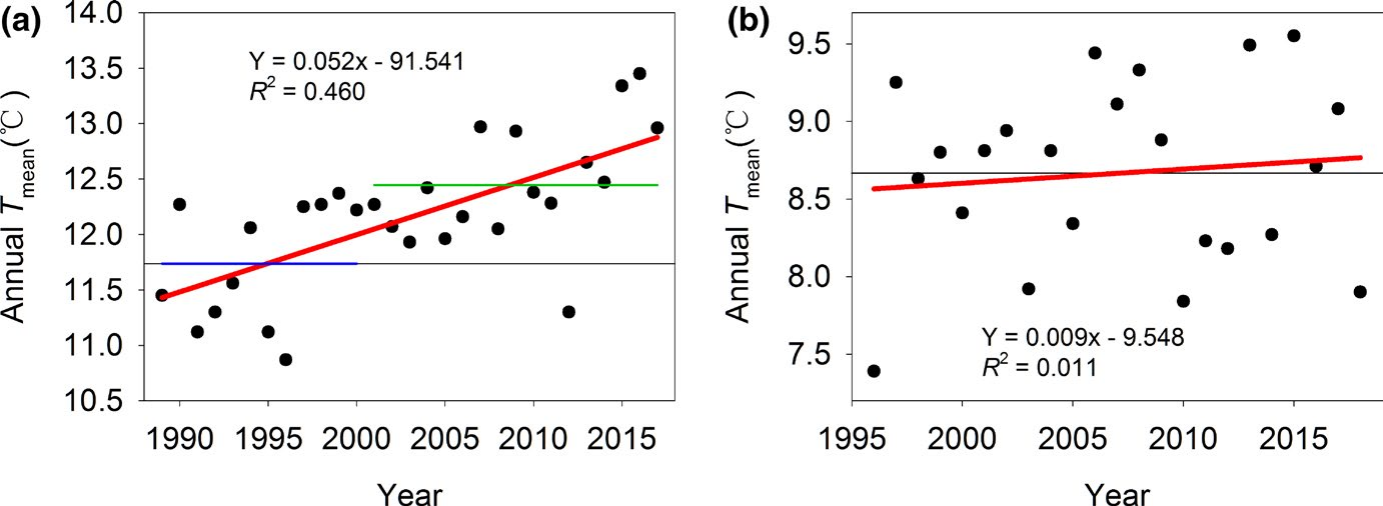
Change trends of annual *T*mean in Maigaiti (a) and Shawan (b). Blue and green solid lines mean the annual *T*mean before and after abrupt change (a), respectively. The abrupt change years in Maigaiti and Shawan were in 2000 and 2010, respectively. The black thin horizontal lines were the mean values

The annual *T*_mean_ in Maigaiti County from 1989 to 2017 increased by 0.052°C per year with significance (Figure 2a), while the annual *T*_mean_ in Shawan County from 1996 to 2018 increased by 0.009°C per year without significance (Figure 2b). The annual *T*_mean_ in Maigaiti and Shawan all showed increasing trends, and the years of abrupt change were 2000 (Figure 2a) and 2010 (Figure 2b), respectively. The annual *T*_mean_ before abrupt changes in Maigaiti and Shawan were 11.74°C and 8.66°C, respectively (Figure 2), while after abrupt change, they were 12.45°C and 8.68°C, respectively (Figure 2). This finding indicated that the annual *T*_mean_ showed a dramatic rise of approximately 0.71°C in Maigaiti but an increase of only approximately 0.02°C in Shawan after the abrupt change. These results showed that the temperature increase in Maigaiti was more significant and stronger than that in Shawan.

### 2.3 Statistical analysis

Adult *H*. *armigera* were trapped during the period from the first appearance date (FD) of adult moths to their appearance end date (ED). The period between the FD and ED of each generation was considered the PD. Every generation was established by analyzing the patterns of population fluctuation, that is, the number of adults trapped by the light trap during the season (Lu & Baker, 2013; Miao et al., 2006).

To illustrate the relationship between moths and accumulated heat, AT was used. AT in one generation meant the sum of daily temperature during the PD, for example, the AT in G0. The total AT represented the sum of AT in G0, G1, and G2 (and G3). The mean temperature in one generation represented the average daily temperature during the PD, for example, *T*_mean_ in G0. The AT percentage in one generation indicated the AT in one generation that accounted for the total AT, for example, the AT percentage in G0 in Maigaiti was 22.8%, which accounted for 22.8% of the total AT in Maigaiti. Similarly, the percentage of adult moths of one generation accounted for the whole number of G0, G1, and G2 (and G3).

The nonparametric Mann–Kendall test (MK) was first developed by Mann (1945) and further developed by Kendall (1948) and Gerstengarbe and Werner (1999). The Mann–Kendall test presents the advantages of a broad detection range, small artificial impact, and high degree of quantitativeness (Wei, 2007). In this study, the Mann–Kendall test procedure with a 5% significance level was applied to analyze abrupt changes according to Gerstengarbe and Werner (1999). To show the effects of climate abrupt change on moths, an analysis of abrupt changes in phenology, number of moths, and AT was employed.

Only the figure over the significant level of 5% was shown. The regression analysis was conducted with SPSS software (SPSS 17.0 for Windows, SPSS Inc., Chicago, IL, USA), and graphs were drawn with SigmaPlot software (SigmaPlot 12.5 for Windows).

## 3 RESULTS

### 3.1 Effects of climate change on different geographical populations

For the moths in G0, G1, and G2, the change in the rate of phenological shifts in Shawan was faster compared with that in Maigaiti, except for the ED of G0 (Table 1). All the FDs were advanced, only the EDs were delayed in G1 at both sites, and all the PDs were prolonged except the PD in G1 in Maigaiti, which was shortened by 0.090 days per year. Moths in G3 only were only observed in Maigaiti and not in Shawan, and the ED and PD showed stronger phenological shifts (Table 1). This finding illustrated that climate change had a greater effect on the phenology of *H*. *armigera* in Shawan than in Maigaiti.

**TABLE 1:** Changes in phenology, sizes, and AT of populations in Maigaiti and Shawan

	Maigaiti		Shawan	
	Change rate/year	Trends	Change rate/year	Trends
FD in G0	−0.467*	Advanced	−0.586*	Advanced
ED in G0	0.208*	Delayed	0.203	Delayed
PD in G0	0.358**	Prolonged	0.776**	Prolonged
FD in G1	−0.207	Advanced	−0.259	Advanced
ED in G1	−0.117	Advanced	−0.194	Advanced
PD in G1	−0.090	Shortened	0.064	Prolonged
FD in G2	−0.334*	Advanced	−0.537	Advanced
ED in G2	0.271	Delayed	0.547	Delayed
PD in G2	0.605**	Delayed	1.084*	Delayed
FD in G3	−0.099	Advanced	——	——
ED in G3	0.516	Delayed	——	——
PD in G3	0.614**	Prolonged	——	——
No. of G0	6.352**	Increased	8.608	Increased
No. of G1	21.207	Increased	10.331	Increased
No. of G2	18.467	Increased	27.682	Increased
No. of G3	21.312	Increased	——	——
Total No.	67.339	Increased	46.621	Increased
AT in G0	15.633**	Increased	16.016*	Increased
AT in G1	2.883	Increased	1.484	Increased
AT in G2	16.763**	Increased	25.799*	Increased
AT in G3	12.946**	Increased	——	——
Total AT	48.225**	Increased	43.299**	Increased

Note

“——” represents that there is no this generation adults in Shawan. G0: overwintering generation adults. G1: the first‐generation adults. G2: the second‐generation adults. G3: the third‐generation adults. FD: the first appearance date. ED: the end appearance date. PD: the period days mean from the first appearance date to the end appearance date. AT: accumulated temperature. *p < .05, **p < .01, and ***p < .001. For the “FD, ED and PD,” the “change rate” means “changes in the rates of phenology shift of FD, ED and PD”; and “Trends” means “advanced, delayed or prolonged of phenology.” For the “No. of G0, G1, G2 and G3, and Total No.,” the “change rate” means “changes in the rates of the number of G0, G1, G2 and G3, and total number”; and “Trends” means “increased or decreased of number of moth.” For the “AT and Total AT”, the “change rate” means “changes in the rates of AT of G0, G1, G2 and G3, and total AT”; and “Trends” means “increased or decreased of AT.”

The changes in the number of adult moth shifts in G0 and G2 in Shawan were faster than those in Maigaiti (Table 1). However, the changes in the number of total moths in Maigaiti and Shawan increased by 67.339 and 46.621 per year, respectively (Table 1). Only the change in the number of G0 moths was significant. These results showed that climate change had a greater effect on the number of moths in Maigaiti than in Shawan.

The changes in the rate of AT shift in G0 and G2 in Shawan were faster than those in Maigaiti (Table 1). Moreover, the changes in the rate of total AT shift in Maigaiti and Shawan rose by 48.225 and 43.299 per year, respectively (Table 1). This result suggested that both sites had almost equal increases in the rates of total AT during PD. Only the changes in the number of G1 moths at both sites were insignificant, while the other changes were all significant.

### 3.2 Relationship between the number of moth and AT

**FIGURE 3:**
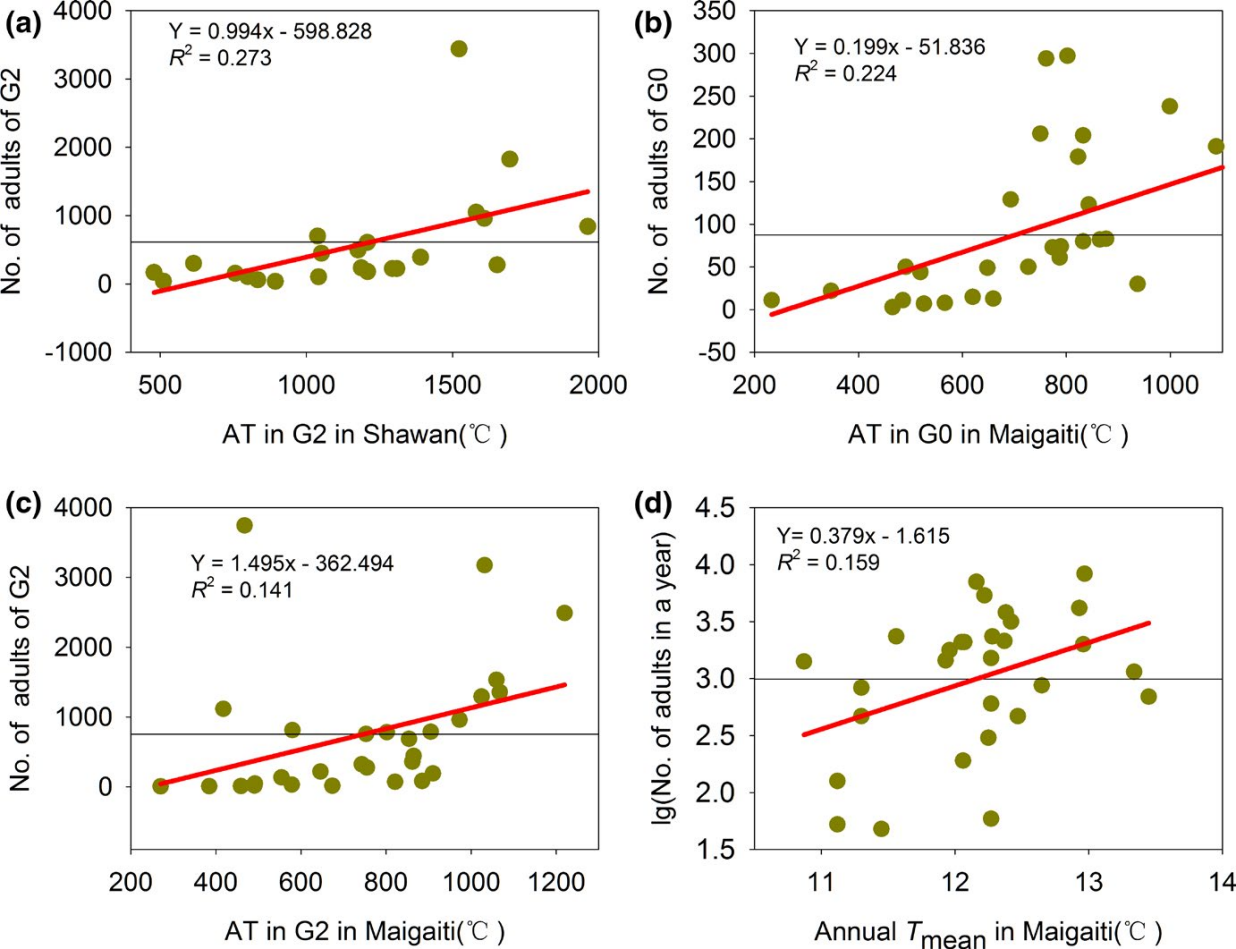
Relationships between the moth number of generations and ATs in Shawan and Maigaiti (a–c), and the relationship between the log value of cotton bollworm adults in a year and annual *T*mean in Maigaiti (d). The black thin horizontal lines were the mean values

For an increase in the AT by 1°C, the changes in the number of moth shifts at both sites were shown in Table 2. For the effects of AT, the number of moths in G1 (Figure S1a) and G2 (Figure 3c) in Maigaiti increased faster compared with that in Shawan (Figure S2b, Figure 3a), and only the number of moths in G0 in Shawan (Figure S2a) showed a slower increase compared with that in Maigaiti (Figure 3b). For the effects of annual *T*_mean_, the increase in the number of total moths in Maigaiti (Figure 3d) was faster than that in Shawan (Figure S3). These results showed that the AT and annual *T*_mean_ had greater effects on the number of moths in Maigaiti than in Shawan.

**TABLE 2:** The changes in the number of moth shifts at both sites for a 1°C increase in the AT

Sites	G0	G1	G2	G3
Shawan	0.235	0.241	0.994*	/
Maigaiti	0.199**	0.435	1.495*	−0.534

Note

*p < .05, **p < .01, and ***p < .001.

**FIGURE 4:**
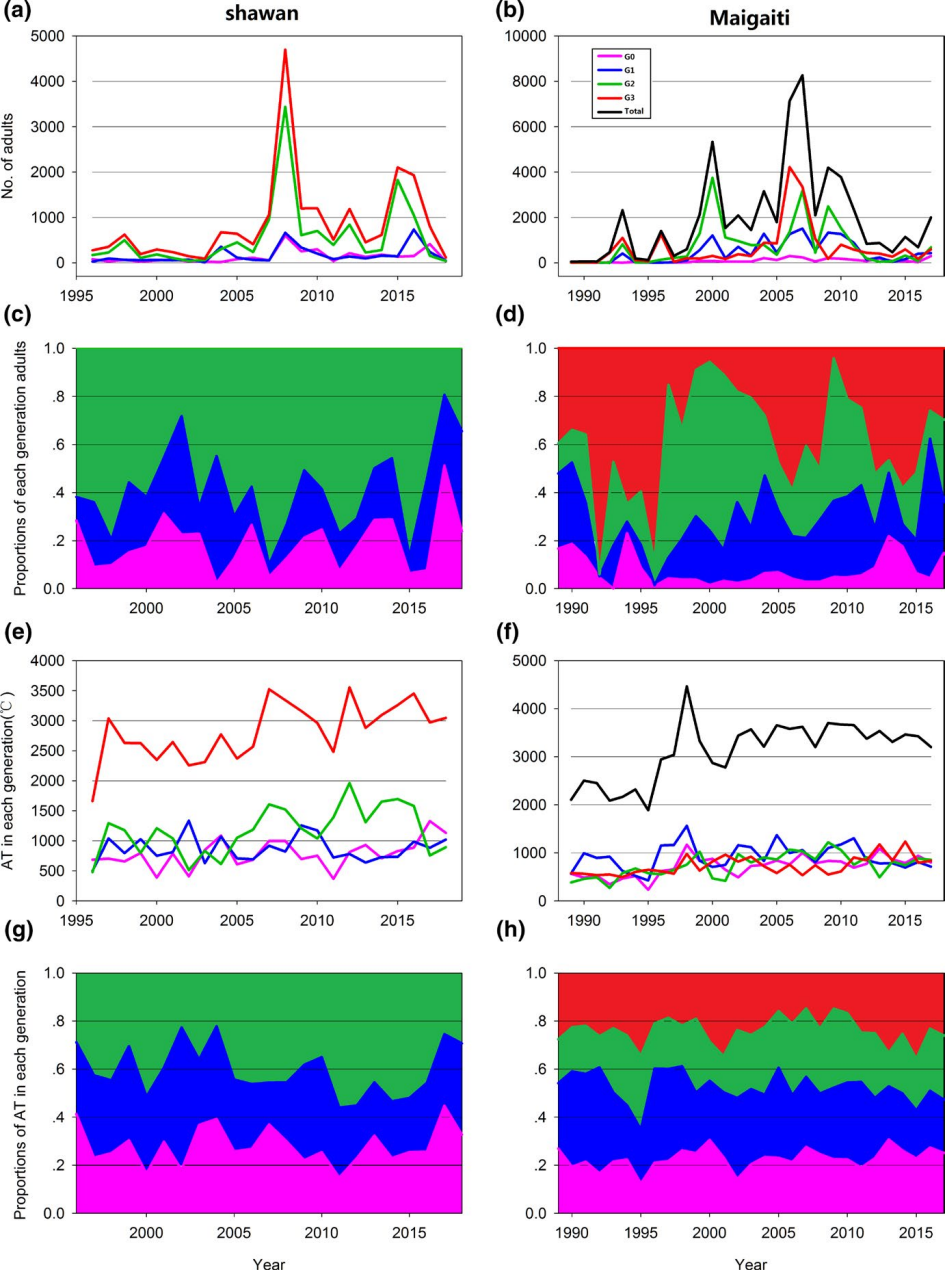
Number, percentages of moth number, AT and percentages of AT of each generation moth in Shawan (a, c, e, g) and Maigaiti (b, d, f, h), respectively

The total number of moths in a year at both sites varied sharply (Table 3; Figure 4a,b). The number of each generation also sharply fluctuated interannually, for example, the fluctuation in the number of moths in G2 (Table 3). However, the percentage of each generation to the total number of individuals in a year was different for the two sites (Table 4; Figure 4c,d). The proportions of G2 in Shawan and G3 in Maigaiti had the largest contribution to the number of local moths.

**TABLE 3:** The changes in the number of total moths and G2 at both sites

Sites	Minimum	Maximum	Mean
Shawan	92	2,103	860.83
`Maigaiti	48	8,271	1,960.76
G2 in Shawan	39	3,439	560.43
G2 in Maigaiti	4	3,745	747.79

**TABLE 4:** The percentage of each generation to the total number of individuals in a year

Sites	G0	G1	G2	G3
Shawan	18.2%	22.6%	58.6%	/
Maigaiti	7.6%	22.0%	31.8%	38.5%

**FIGURE 5:**
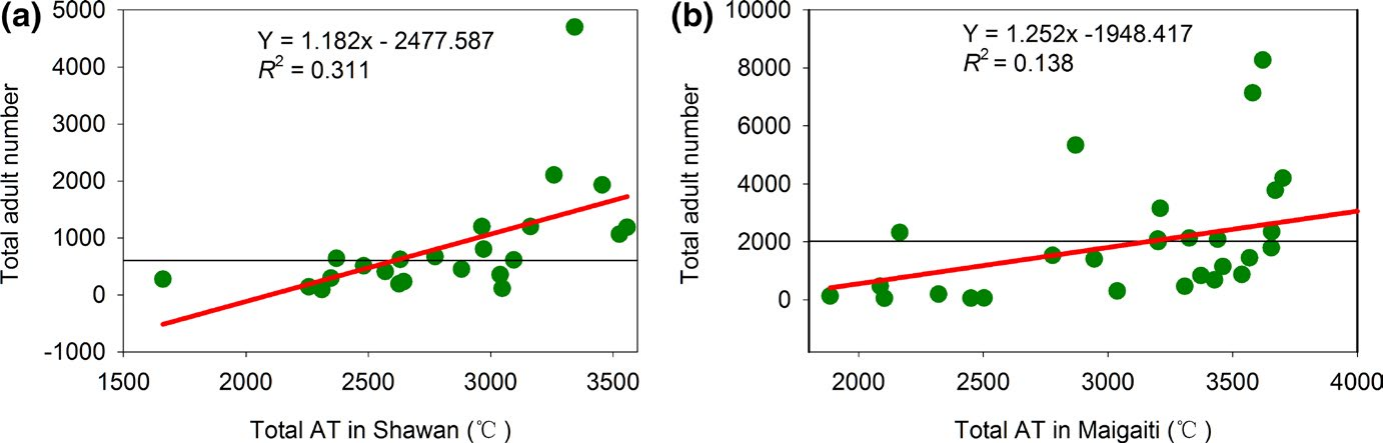
Relationship between total moth number and total AT. The black thin horizontal lines were the mean values

An appropriate temperature and AT are required for the growth of *H*. *armigera*. The AT of each generation and total AT both sites varied significantly (Table 5; Figure 4e,f). Accordingly, the mean number of total moths in Shawan and Maigaiti was 860.83 and 1960.76, respectively (Figure 4a,b). The AT significantly correlated with the number of moths. Furthermore, the total AT in a year increased by 1°C and the number of moths in Shawan and Maigaiti increased by 1.365 and 1.811, respectively, which was significant (Figure 5a,b). This finding further illustrated that climate warming would help increase the number of moths of *H*. *armigera*.

**TABLE 5:** The mean AT of each generation and total AT both sites (°C)

Sites	G0	G1	G2	G3	Total
Shawan	787.57	870.86	1,166.06	/	2,428.49
Maigaiti	720.99	919.70	742.79	738.98	3,122.47

**FIGURE 6:**
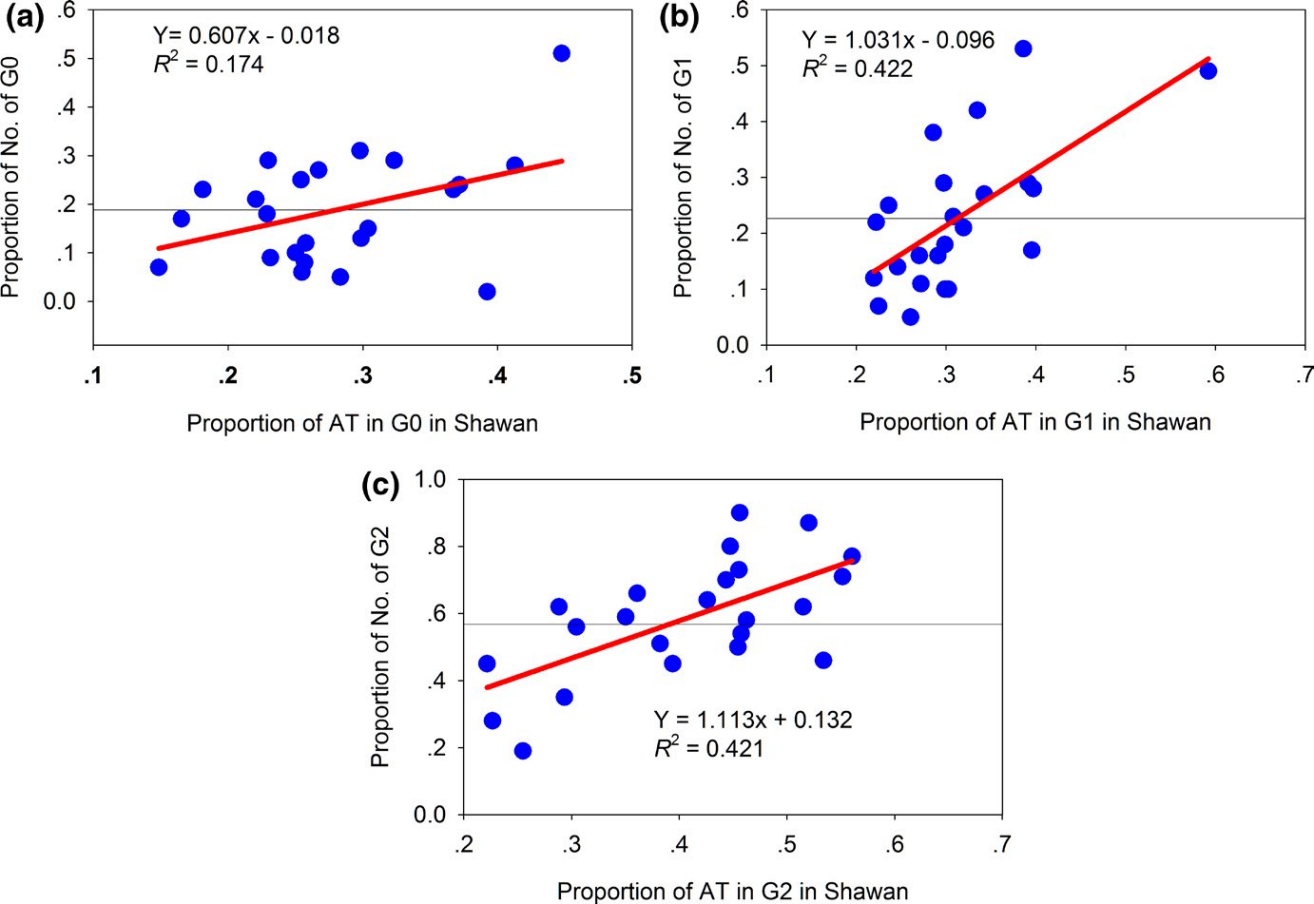
Relationships between the proportions of moth and the proportions of AT. The black thin horizontal lines were the mean values

The mean percentages of AT in each generation at both sites fluctuated (Table 6). The increases in the percentages of moths with a 1% increase in AT in each generation at both sites were different (Table 7; Figure 6). However, only the relationships in Shawan were significant (Figure 6a‐c). The changes in the rates of the number of G0, G1, and G2 in Shawan were faster than those in Maigaiti (Figure. S4a–c), and the change in the rate of the number of G1 in Maigaiti exhibited negative growth (Figure S4b). These results indicated that an increase in AT produced different effects on different generations.

**TABLE 6:** The mean percentages of AT in each generation at both sites

Sites	G0	G1	G2	G3
Shawan	28.0%	31.3%	40.7%	/
Maigaiti	22.8%	29.5%	23.6%	24.1%

**TABLE 7:** The increase in the percentages of moths with a 1% increase in AT in each generation at both sites

Sites	G0	G1	G2	G3
Shawan	0.6%*	1.0%**	1.1%**	/
Maigaiti	0.3%	−0.2%	0.3%	0.7%

### 3.3 Analysis of abrupt changes in the phenology, number of moths, and AT

**FIGURE 7:**
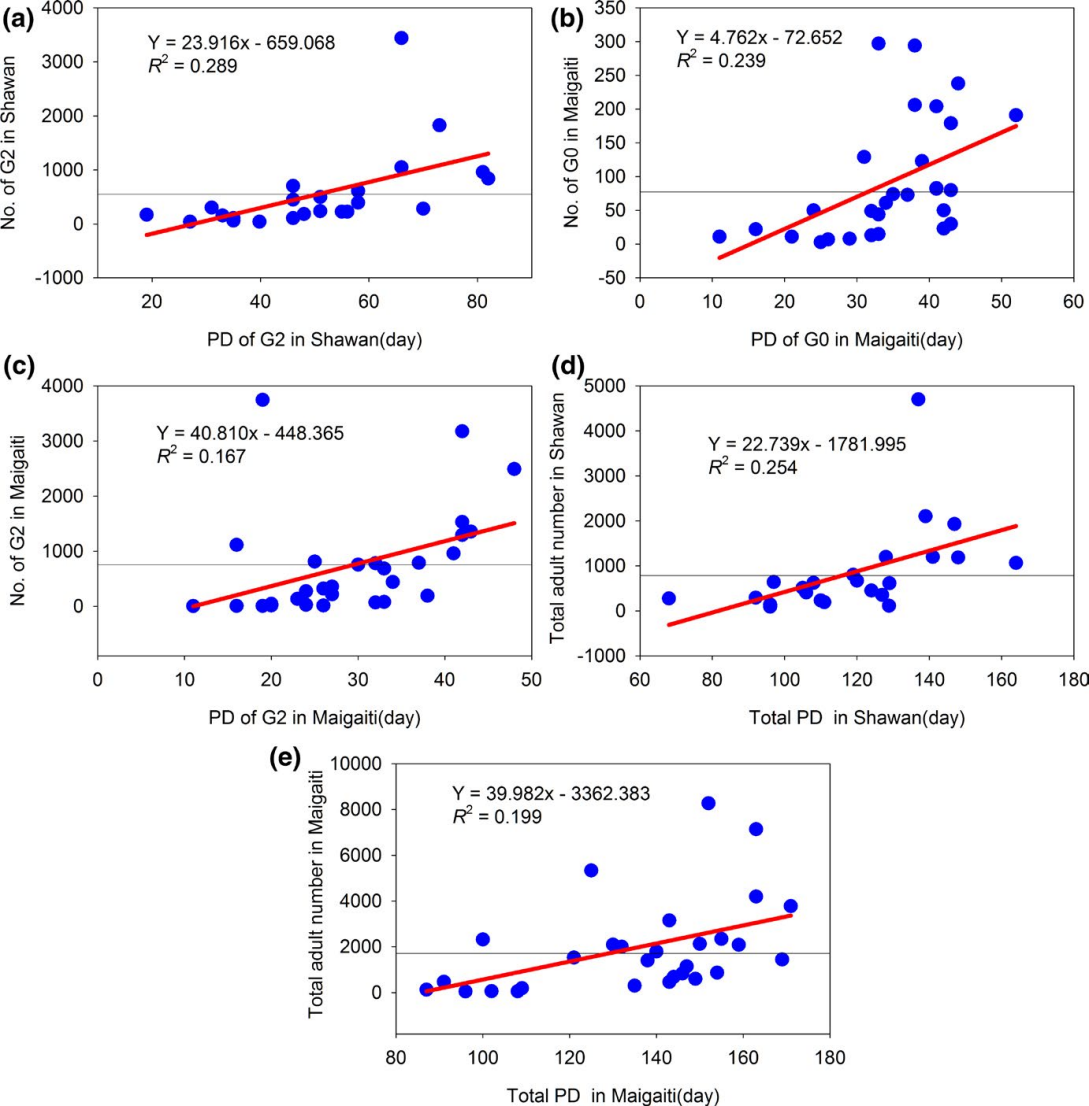
Relationships between the moth number and the PD. The black thin horizontal lines were the mean values

The abrupt changes in ED and PD in G1 in Maigaiti and PD in G1 in Shawan were insignificant, while the abrupt changes in the other phenologies at both sites were significant (Table 8). After abrupt change, the trends were advanced, delayed, or prolonged. The FDs of G0, G1, G2, and G3 all showed advancing trends, and the abrupt change years of FD for G0, G1, and G2 in Maigaiti were all earlier than those in Shawan. The EDs of G0 and G2 in Maigaiti and G2 in Shawan showed delaying trends, while the other EDs showed advancing trends. The EDs of G1 and G2 in Maigaiti were earlier than those in Shawan, and only the abrupt change in ED of G0 was later in Maigaiti than in Shawan. The PDs of G0, G1, and G2 all showed prolonging trends (Figure 7a–c, Figure S5a–c), and only the PD of G3 in Maigaiti showed a shortening trend (Figure S5d). The years of abrupt change in PD in Maigaiti were earlier than those in Shawan. All the abrupt change years of phenology in Maigaiti were earlier than those in Shawan except the ED of G0 (Table 8). These results illustrated that climate warming advanced FD, delayed ED, and prolonged PD. Even if the ED was advanced, the advancing range of FD was greater than the delaying range of ED; therefore, the PD was prolonged. Moreover, only the change in PD of G3 in Maigaiti was insignificant; therefore, all the PDs were prolonged. A longer PD corresponded to a greater number of moths. Because the total PD increased by 1 day in a year, the number of total moths in Shawan and Maigaiti increased by 22.739 (Figure 7d) and 39.982 (Figure 7e), respectively. This finding also illustrated that climate change had different effects on different geographical populations, and an earlier abrupt change corresponded to a greater number of *H*. *armigera* moths.

**TABLE 8:** Abrupt changes in phenology, numbers of adults, and AT in Maigaiti and Shawan

	Maigaiti		Shawan	
	Abrupt change	Trend after	Abrupt change	Trend after
	Year	Abrupt change	Year	Abrupt change
FD in G0	1995*	Advanced	2006*	Advanced
ED in G0	2013*	Delayed	2005*	Advanced
PD in G0	1997*	Prolonged	2015*	Prolonged
FD in G1	1993*	Advanced	2006*	Advanced
ED in G1	1996	Advanced	2007*	Advanced
PD in G1	1995	Prolonged	2011	Prolonged
FD in G2	2001*	Advanced	2007*	Advanced
ED in G2	1991*	Delayed	2000*	Delayed
PD in G2	1994*	Prolonged	2006*	Prolonged
FD in G3	2014*	Advanced	_____	_____
ED in G3	1993*	Advanced	_____	_____
PD in G3	1997	Prolonged	_____	_____
No.in G0	1998*	Increased	2006*	Increased
No.in G1	1998*	Increased	2004*	Increased
No.in G2	1992*	Increased	2004*	Increased
No.in G3	1995*	Increased	_____	_____
Total No.	1992*	Increased	2005*	Increased
AT in G0	2003*	Increased	2012	Increased
AT in G1	1996*	Increased	2002	Increased
AT in G2	1994*	Increased	2006*	Increased
AT in G3	1997*	Increased	_____	_____
Total AT	2000*	Increased	2010	Increased

Note

“——” represents that there is no this generation adults in Shawan. G0: overwintering generation adults. G1: the first‐generation adults. G2: the second‐generation adults. G3: the third‐generation adults. FD: the first appearance date. ED: the end appearance date. PD: the period days mean from the first appearance date to the end appearance date. AT: accumulated temperature. Total AT: the sum of AT in G0, G1, and G2 (and G3). *p < .05, **p < .01, and ***p < .001*.

In Maigaiti, all the abrupt change years of the number of *H*. *armigera* moths in each generation and the total number of moths occurred earlier compared with Shawan (Table 8). This finding illustrated that the increasing trend of *H*. *armigera* moths occurred earlier in Maigaiti than in Shawan. Both sites showed increasing trends, which explained why an increasing number of *H*. *armigera* moths would appear at both sites.

In Maigaiti, all the abrupt change years of AT occurred earlier compared with Shawan (Table 8). Both sites showed increasing trends, and the increasing trend in Maigaiti was earlier than that in Shawan. The effect of climate warming on Maigaiti was greater than that in Shawan.

## 4 DISCUSSION

Climate change led to different dynamics in the population sizes. The mean number of *H*. *armigera* moths in G0 in Shawan and Maigaiti was 132.87 and 91.38, respectively. Maigaiti had fewer moths and a lower moth percentage in G0 than Shawan. One of the reasons was that the snow depth in winter was below 5 cm in Maigaiti but over 20 cm in Shawan (Zhang & Zhang, 2006), and the deeper snow can provide higher temperatures for *H*. *armigera*, which increases the overwintering survival rate (Huang, 2016, 2017; Huang & Li, 2015). In addition, the ATs of G0 in Shawan and Maigaiti were 787.57°C and 720.99°C, respectively. When the AT increased by 1°C, the number of moths in the G0 in Shawan and Maigaiti increased by 0.235 (Figure S2a) and 0.199 (Figure 3b), respectively. Furthermore, the mean PDs in Shawan and Maigaiti were 34.69 days and 34.45 days, respectively (Table 9). The PD of G0 increased by 1 day, and the number of *H*. *armigera* moths of G0 in Shawan and Maigaiti increased by 4.096 (Figure S5a) and 4.762 (Figure 7b), respectively. This finding illustrated that prolonging PD led to an increased number of *H*. *armigera*. However, the PD values at both sites were almost identical (Table 9); therefore, the increase in the number of *H*. *armigera* moths caused by PD was not the major factor underlying the differences. Thus, the differences in AT and snow depth between both sites led to the differences in the number of *H*. *armigera* moths in G0.

**TABLE 9:** The mean value of each generation in Maigaiti (1989–2017) and Shawan (1996–2018)

	Maigaiti	Shawan
No. of G0	91.38	132.87
No. of G1	466.17	167.52
No. of G2	747.79	560.43
No. of G3	655.41	——
*T*_mean_ of G0 (°C)	20.92	22.71
*T*_mean_ of G1 (°C)	24.73	26.08
*T*_mean_ of G2 (°C)	25.38	22.83
*T*_mean_ of G3 (°C)	21.52	——
AT of G0 (°C)	720.99	787.57
AT of G1 (°C)	919.70	870.86
AT of G2 (°C)	742.79	1,166.06
AT of G3 (°C)	738.98	——
PD of G0 (day)	34.45	34.69
PD of G1 (day)	37.03	33.49
PD of G2 (day)	29.31	50.99
PD of G3 (day)	34.45	——
Proportion of G0 No. (%)	7.63	18.82
Proportion of G1 No. (%)	22.03	22.63
Proportion of G2 No. (%)	31.82	58.55
Proportion of G3 No. (%)	38.52	——
Proportion of G0 AT (%)	22.82	28.02
Proportion of G1 AT (%)	29.50	31.28
Proportion of G2 AT (%)	23.62	40.70
Proportion of G3 AT (%)	24.06	——

The mean numbers of *H*. *armigera* moths in G1 in Maigaiti and Shawan were 466.17 and 167.52 (Table 9), respectively, and the number of G1 moths in Maigaiti and Shawan increased by approximately 4.10 and 0.26 times compared with the number of G0 moths, respectively. The AT and PD led to these results. The ATs of G1 in Maigaiti and Shawan were 919.70°C and 870.86°C, respectively. For an increase in the AT by 1°C, the number of G1 moths in Maigaiti and Shawan increased by 0.435 (Figure S1a) and 0.241 (Figure S2b), respectively. Furthermore, the mean PDs of G1 in Maigaiti and Shawan were 37.03 days and 33.49 days, respectively (Table 9). The PD increased by 1 day, and the number of *H*. *armigera* moths of G1 in Maigaiti and Shawan increased by 16.368 (Figure S5c) and 7.356 (Figure S5b), respectively. Thus, the higher AT and the longer PD in Maigaiti led to a dramatic increase in the number of the *H*. *armigera* moths in G1. Similarly, the number of G2 moths in Maigaiti and Shawan increased by approximately 0.60 and 2.35 times compared with the number of G1 moths, respectively. Because the Shawan population of G2 had a higher AT and longer PD (Table 9), the moths in Shawan increased dramatically. Thus, climate change had different effects on the number of *H*. *armigera* moths of different generations, and the major affecting factors were different for each generation.

Many studies have indicated that increased AT increases the number of insects (Bartekova & Praslicka, 2016; Jallow et al., 2001; Qureshi et al., 1999); however, this view was not supported by the number of *H*. *armigera* moths in G3 in Maigaiti (Figure S1b). Approximately half of time in G3 was in autumn, when the air temperature decreased and diapause began. When the air temperature is 22°C and sunshine hours are below 13 h and 22 min, *H*. *armigera* begins diapause (Wu & Guo, 1995). Among the 999 days of G3 from 1989 to 2017 in Maigaiti, there were 592 days whose daily mean air temperature was below 22°C. In addition, air temperature of 21°C can inhibit the production of ecdysone by prothoracic glands (Meola & Adkisson, 1977), and a lower content of ecdysone leads to *H*. *armigera* diapause (Wang & Gong, 1997). Furthermore, pupal diapause is induced at 20°C by short photoperiods and inhibited by long photoperiods during the larval stage, and diapause is induced at 15°C regardless of photoperiod (Kurban et al., 2007). These factors accelerated the *H*. *armigera* diapause process. Thus, pupae in diapause led to reduced moth emergence, which led to a slow increase in the number of moths. With prolongation of PD for G3, the air temperature became increasingly lower and an increasing number of diapause pupae were produced, which led to the increasingly less frequent emergence of moths. Therefore, with prolonged PD for G3 in Maigaiti, the increase in the rate of the number of moths was negative (Table 9). Thus, when considering the influence of AT on *H*. *armigera* moths, the life cycle of *H*. *armigera* should be considered.

Significant differences in pest pressure were observed between the sites, which were corroborated by the average number of trapped specimens and the regression coefficients (Figures 3 and 4). In addition to climate factors, the crop planting structure was another important influencing factor. When the planting percentage of non‐*Bt* cotton is over 50%, a substantial increase in cotton bollworm number may be observed (Huang & Hao, 2020); thus, the number of *H*. *armigera* moths in Maigaiti sharply increased. However, according to Lu et al. (2012), the moths in Shawan developed in a complex landscape that corresponds to a greater production of moths compared with that in a simple landscape because insects can feed on many kinds of plants to avoid lack of food (Allen & Luttrell, 2009; Maelzer & Zalucki, 1999; Slosser et al., 1987). However, there were fewer *H*. *armigera* moths in Shawan than in Maigaiti due to many factors.

The percentages from G0 to G2 or G3 at both sites increased (Table 9). Crop damage caused by *H*. *armigera* was mainly observed during G2 and G3 at both sites, and the total percentage of G2 and G3 in Maigaiti was 70.3%, while the percentage of G2 in Shawan was 58.5%. Obviously, the damage caused by *H*. *armigera* in Maigaiti was greater than that in Shawan. Meanwhile, the AT percentages of G2 and G3 in Maigaiti increased by 0.2% and 0.0% per year, respectively, which meant that the AT percentage of G3 would not increase. The AT percentage of G2 in Shawan increased by 0.3% per year. When the AT percentage increased by 1%, the *H*. *armigera* moths in Maigaiti and Shawan increased by 23.62 and 40.70, respectively (Table 9). Thus, might suggest that the higher increase in the AT percentage of G2 in Shawan led to the production of more moths compared with that in Maigaiti, which would lead to greater damage in Shawan that might be much heavier than that in Maigaiti. Such a dynamic might indicate that the damage to crops caused by *H*. *armigera* would be greater at higher latitudes than lower latitudes under climate warming.

An abrupt change in temperature usually precedes an abrupt phenological change (Liu et al., 2007). Most of the phenological abrupt changes at both sites occurred according to the rule, and only the abrupt changes in ED in G0 and FD in G3 in Maigaiti and PD in G0 and G1 in Shawan were later than that of the annual *T*_mean_ at both sites (Table 8). However, the abrupt change years of phenology, AT, and the number of *H*. *armigera* moths occurred earlier in Maigaiti than Shawan except for ED in G0 (Table 8). This finding explains why the abrupt change year for the annual *T*_mean_ was earlier in Maigaiti than Shawan (Figure 2). In fact, after an abrupt change, the annual *T*_mean_ dramatically increased by approximately 0.71°C in Maigaiti but only increased by approximately 0.02°C in Shawan (Figure 2). Abrupt climate change led to increases in the mean temperature, which would further affect the development, phenology, and numbers of *H*. *armigera*. In recent decades, the annual *T*_mean_ in Maigaiti increased dramatically compared with that in Shawan (Figure 3h,i), which caused *H*. *armigera* in Maigaiti to be much more abundant than that in Shawan. These factors caused the differences between the two populations at both sites.

## 5 CONCLUSIONS

Our results suggested that (a) climate change had a greater effect on the phenology of *H*. *armigera* at higher latitudes than at lower latitudes; (b) climate change caused a greater increase in population size at lower latitudes than at higher latitudes; (c) the AT percentage had a more rapid increase at higher latitudes than lower latitudes and will cause increases in adult moths; and (d) the abrupt changes in phenology, AT, and population size at lower latitudes occurred earlier than those at higher latitudes. Climate change had different effects on different geographical populations and even on different generations. For each generation, the major influencing factors were different. Thus, it is necessary to develop sustainable management strategies for *Helicoverpa armigera* at an early stage.

## CONFLICT OF INTEREST

None declared.

## AUTHOR CONTRIBUTION

**Jian Huang:** Conceptualization (lead); data curation (lead); formal analysis (lead); funding acquisition (lead); investigation (lead); methodology (lead); project administration (lead); resources (lead); software (lead); supervision (lead); validation (lead); visualization (lead); writing – original draft (lead); writing – review and editing (lead).

## Supporting information

Appendix S1

## Data Availability

The data can be found in Figshare. https://figshare.com/account/home. https://doi.org/10.6084/m9.figshare.14822733
