# Supplementary material for: Effects of climate change on different geographical populations of the cotton bollworm Helicoverpa armigera (Lepidoptera, Noctuidae)
Source: Ecol Evol. 2021 Dec 6;11(24):18357–68. doi: 10.1002/ece3.8426 (PMC8717297; doi:10.1002/ece3.8426)
Supplement: Supplementary file 1 — Appendix S1 [file ECE3-11-18357-s001.docx]

**Appendix**


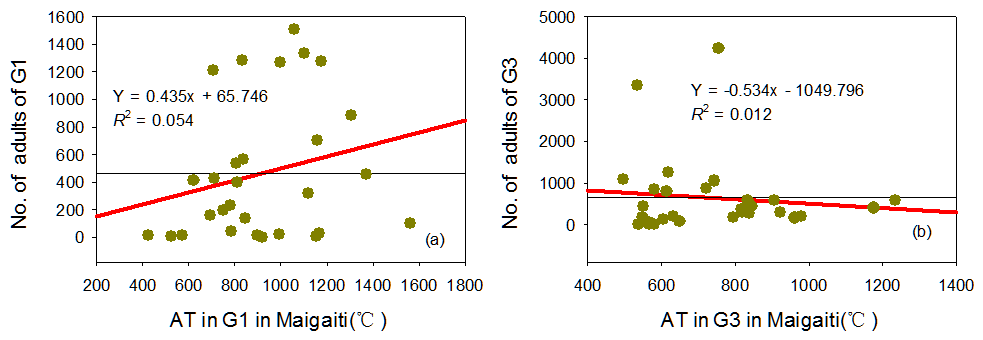


**Fig. S1 The relationship between AT and adults of G1 and G3 in Maigaiti.** **The black thin horizontal lines were the mean values.**


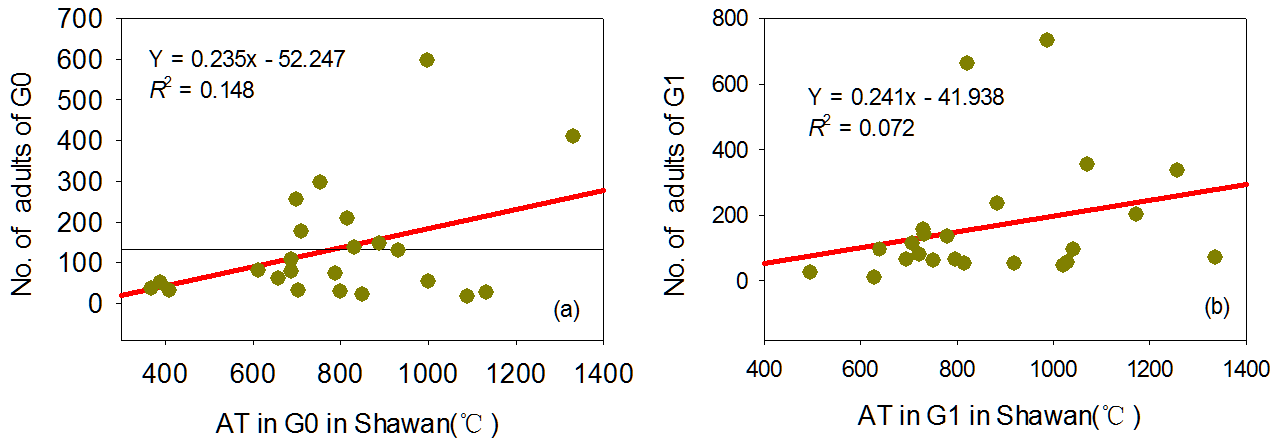


**Fig. S2 The relationship between AT and adults of G0 in Shawan.** **The black thin horizontal lines were the mean values.**

**
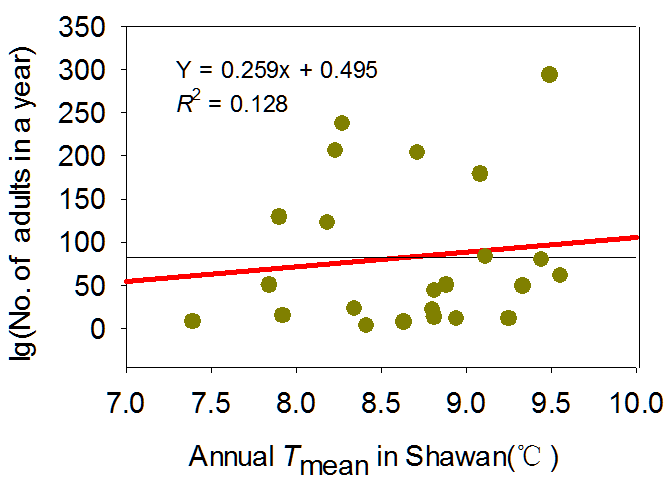
**

**Fig. S3 The relationship between annual *T*_mean_ and adults in a year in Shawan.** **The black thin horizontal lines were the mean values.**

**
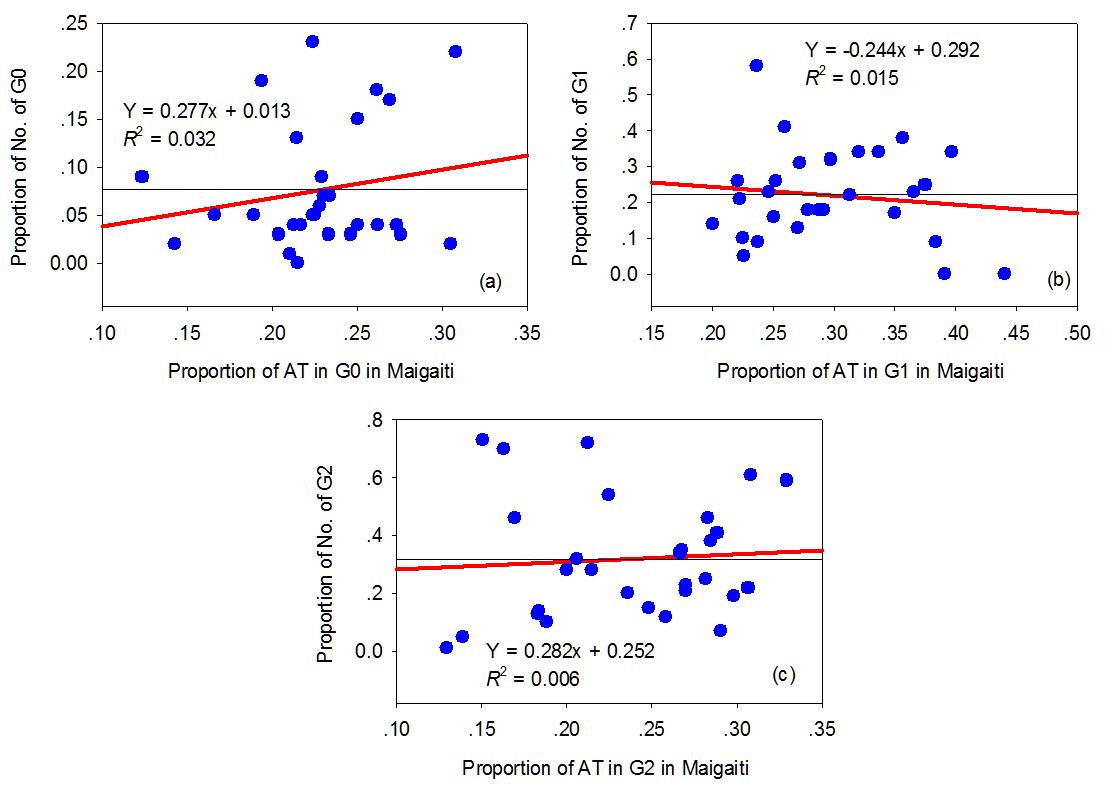
**

**Fig. S4 Relationships between the proportions of moth and the proportions of AT in Maigaiti. The black thin horizontal lines were the mean values.**

**
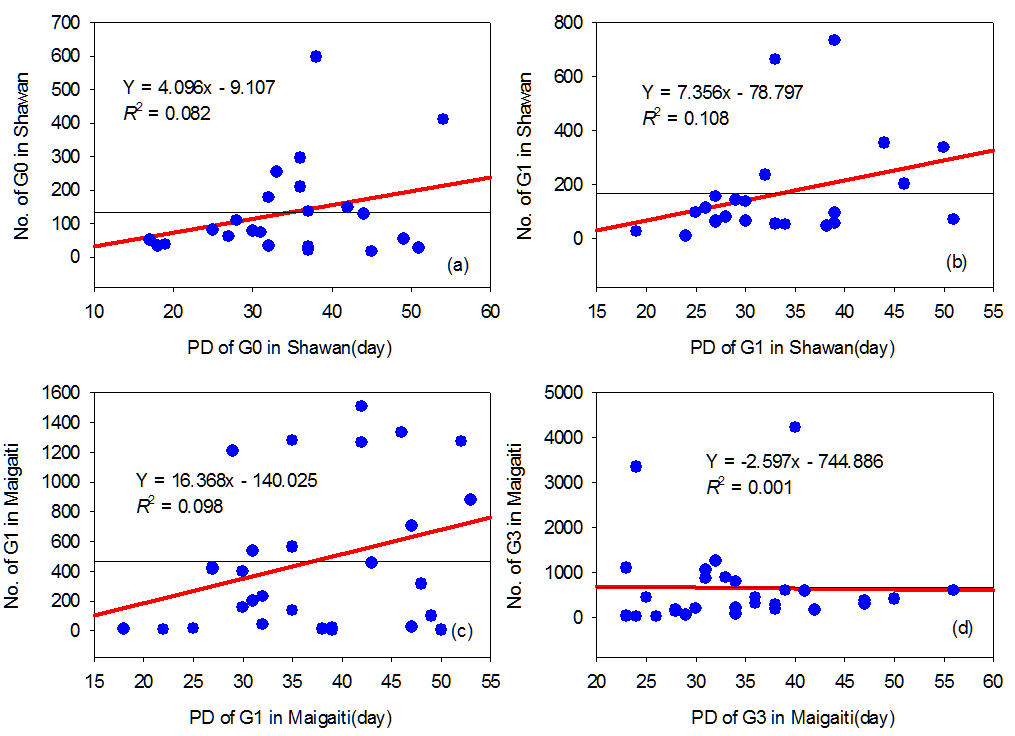
**

**Fig. S5 Relationships between the moth number and the PD. The black thin horizontal lines were the mean values.**
